# Arthroscopic isolated posterior labral repair in rugby players

**DOI:** 10.4103/0973-6042.50875

**Published:** 2009

**Authors:** Ravi Badge, Amol Tambe, Lennard Funk

**Affiliations:** Upper Limb Unit, Wrightington, Wigan and Leigh NHS Trust, Appley Bridge, WN6 9EP, UK

**Keywords:** Arthroscopic repair, contact athletes, posterior labral injury, return to sport, rugby

## Abstract

**Background:**

The shoulder is the second most frequently injured joint after the knee in rugby players and labral tears appear to be common. There is limited data available in the literature regarding the mechanisms of posterior labral injury in rugby players and the management of these injuries.

**Objective:**

The aim of this study is to report the clinical presentation, arthroscopic findings, surgical technique for repair, and the functional outcome in elite English rugby players with isolated posterior labral injuries.

**Study Design:**

Case series (level IV evidence)

**Materials and Methods:**

Over a 5-year period we surgically treated 142 elite rugby players, of whom 11 (7.8%) had isolated posterior labral injuries. All these 11 patients had significant contact injury. Only three (24%) patients had a true posterior shoulder dislocation. Pre- and postoperative assessment included Constant score, Oxford shoulder score, and Oxford instability score. We also assessed the time taken to return to preinjury level of fitness and the complications of surgery.

**Results:**

Average follow-up was for 32 months (range 17–54 months). The mean Constant score improved from 66 to 99. The Oxford score indicated improvement, decreasing from 33 to 18; similarly, the Oxford instability score also decreased from 52.2 to 12.3. Return to playing rugby at peak level was at a mean of 4.3 months after arthroscopic repair.

**Conclusion:**

Successful clinical results and rapid return to play can be achieved by appropriate early arthroscopic repair and supervised accelerated rehabilitation for posterior labral tears in elite rugby players.

## INTRODUCTION

Rugby is one of the most popular contact sports in the world. It is an extremely physical game and there is a significant incidence of serious injuries. The shoulder is the second most frequently injured joint after the knee in rugby players and thus is responsible for a significant number of player hours lost.[1]

There are few studies in the literature addressing injuries to the posterior labrum in contact athletes,[2,3] and there are none that address this injury in elite English rugby players. This is significant because the playing conditions, as well as some of the rules, in England are different from that in America.

Posterior labral injury is less common than its anterior counterpart. It occurs in approximately 2–5% of all cases of shoulder instability.[4] While there has been an improved awareness and understanding of this injury in the last two decades, it still remains a diagnostic and therapeutic dilemma.[5]

The aim of this study is to investigate and study the results and functional outcome of posterior labral injuries in elite rugby players.

## MATERIALS AND METHODS

We reviewed 142 elite rugby players (professional and semiprofessional) who underwent arthroscopic shoulder surgery by the senior author (L.F.) between 2003 and 2007. The patients were identified from a computerised database. Patients with a documented, isolated posterior labral injury that had been treated by the arthroscopic technique were included in this study. The case notes were studied to collect data on the demographics, mechanism of injury, and presenting complaints. All players had undergone routine preoperative functional scoring and these scores were noted. Operative records were also studied to note the operative technique used. Postoperatively, Constant scores, Oxford shoulder scores, and Oxford shoulder instability scores were performed on all patients; we also assessed patient satisfaction with the results of the treatment and the time taken to return to the sport.

All patients in this study were males; the mean age was 24.8 years (range: 15–36). All patients had sustained a significant shoulder injury either as a result of a tackle or a direct blow during match play. Only three (27%) patients had actual shoulder dislocations on field. Most of the players described a 'pop' in the involved shoulder at the time of the injury or complained of a 'dead arm feeling' after the traumatic episode. The dominant arm was involved in six (55%) patients. All patients had diffuse pain in the involved shoulder, especially during bench pressing. Two patients had had previous surgery on the involved shoulder, of which one was for SLAP (Superior Labral Antero-Posterior) repair and the other being for an anterior stabilization.

On physical examination, five patients had a positive O'Brien test for SLAP tear and only three (27%) had signs of posterior instability. One player did not have any significant positive findings apart from posterior joint-line tenderness. All patients had nearly full range of motion. None of the patients displayed multiligamentous laxity.

One patient had a small posterior bony Bankart lesion in the standard plain radiograph. Six of the eleven patients had undergone MR arthrogram preoperatively and in all cases there were confirmed soft tissue reverse Bankart lesions.

**Figure 1:**
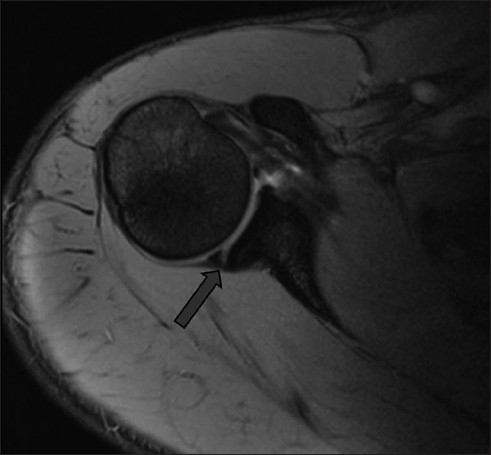
Posterior labral tear as seen on axial MR arthrogram image. The arrow shows the tear

There were a total of 29 (20.4%) patients with posterior labral injuries, of which 11 (7.7%) had isolated posterior labral injury. All patients had undergone a thorough clinical assessment by the senior author in the shoulder clinic. All had had standard anteroposterior and axillary radiographs taken of the involved shoulder and, when indicated, further investigation had been done in the form of MR arthrogram or ultrasound [Figure 1]. Ultrasound had been performed in the clinic whenever there was a strong clinical suspicion of a rotator cuff tear. MR arthrograms were performed on all patients.

We excluded patients with any associated injuries to the anterior labrum and the rotator cuff and also those with less than 1 year of follow-up.

### Surgical technique

**Figure 2:**
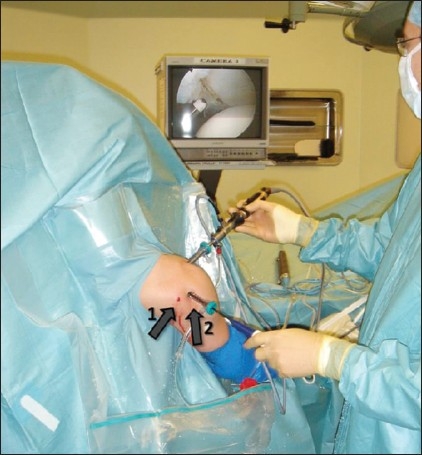
The posterior working portal (2) is more lateral to the standard posterior portal (1). It usually lies 2–3 cm directly below the posterolateral angle of the acromion

**Figure 3:**
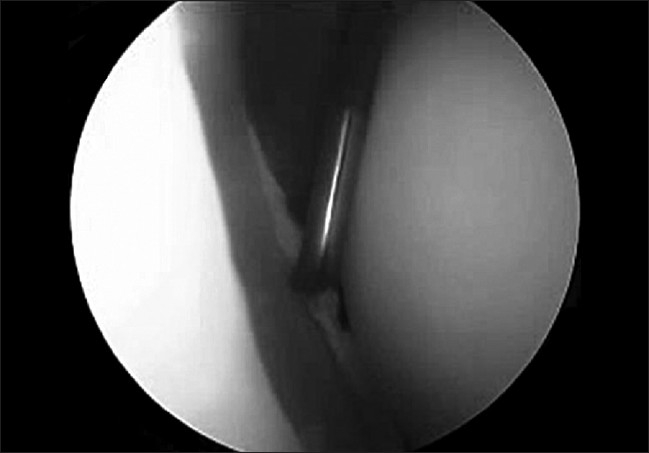
Probing of a posterior labral tear of the left shoulder, as seen from the anterior portal

Surgery was performed in the beach-chair position, using standard arthroscopic equipment and methods. A preoperative examination under anesthesia was routinely performed. The initial posterior portal was made much more lateral than the standard posterior soft-spot portal [Figure 2] to allow for direct access to the posterior glenoid. The anterior and superior structures were visualized from the posterior portal and probed from the anterior portal initially. The scope was then changed to the anterior portal and instrumentation was done via the posterior portal. The extent of the labral injury was confirmed by probing [Figure 3].

**Figure 4:**
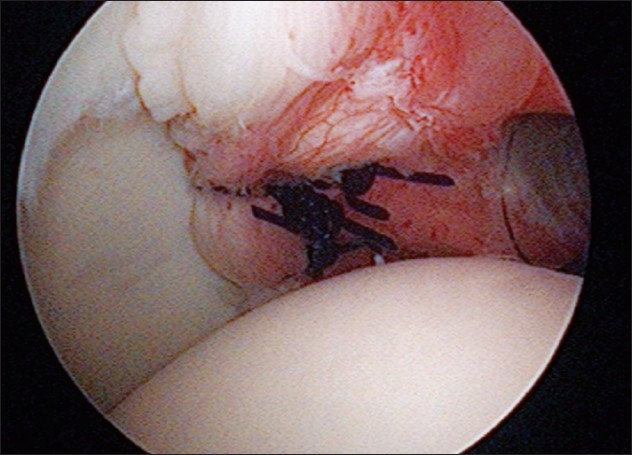
Posterior capsulolabral repair with two suture anchors

The labrum was mobilized and the posterior glenoid rim was prepared. Suture anchor repair was performed with suture anchors, using biodegradable anchors and high-strength sutures [Figure 4]. One to four (mean: 2.3) anchors were used in each case, depending on the size of the tear. An additional posterior capsular plication was added if the posterior capsule was felt to be particularly lax. This was determined by examination under anesthesia, visual inspection, and palpation. Only two patients required this and their outcome or recovery was no different from that of the other cases.

### Postoperative rehabilitation

Initial immobilization was in a 15º external rotation sling for 3 weeks. During this time patients progressed from active assisted mobilization to active mobilization under the supervision of the team's therapists. Closed chain exercises were commenced immediately postoperatively and open chain exercises were added after 3 weeks. Resistance exercises were introduced after approximately 3 weeks and sports-specific rehabilitation was started after 2–3 months. Patients returned to tackle-bags and impact after they had achieved satisfactory movement, strength, isokinetic, and proprioceptive criteria. A strict time limit was not enforced for return to full play, but was decided after a discussion between the therapist, surgeon, player, and the conditioning coach.

## RESULTS

In all patients, thorough examination of both shoulders were performed after anesthesia, once patient was completely relaxed. All shoulders were graded using the scale described by Altchek *et al*. to quantify posterior humeral translation.[6] Examination under anesthesia demonstrated grade I posterior laxity in six and grade II laxity in five cases.

At arthroscopy, 10 (92%) subjects had posterior labral tears (reverse Bankart lesions), while 1 (8%) had a bony reverse Bankart lesion. Labral injury was in the posteroinferior quadrant of the glenoid in nine (81%) patients and in the posterosuperior quadrant in two (19%) patients.

All the patients made uneventful postoperative recovery, without any immediate or early postoperative complications. They were reviewed at regular intervals in the shoulder clinic by the senior author, when Constant, Oxford, and instability scores were also performed. All patients were assessed by experienced shoulder physiotherapists and supervised accelerated shoulder rehabilitation was started immediately postoperatively by the club therapists.

The mean follow-up was 32 months (range 17–54 months). The Constant score improved from a mean of 66 preoperatively to 99 postoperatively. The Oxford score indicated improvement, decreasing from 33 to 18 and, similarly, the Oxford instability score also decreased from 52.2 to 12.3.

The mean time for return to rugby after surgery was 4.3 months (range: 3–6 months). All returned to their previous level of play. At the time of completion of this study, none of the rugby players had had recurrence of symptoms.

One patient had a further tackling injury to the same shoulder the following season and required surgery. Arthroscopy in this patient revealed an anterior labral tear with evidence of an intact and healed previous posterior repair. One patient was diagnosed with a loose suture anchor at 3 months. He had not returned to play at that stage. At arthroscopy, the repair was found to have healed and the anchor was simply removed. He returned to play 4 weeks after anchor removal.

## DISCUSSION

Of all sports, rugby has the highest risk of injury per player- hour. The shoulder is the second most commonly injured joint after the knee, with shoulder injuries constituting 20% of all rugby injuries. Thirty-five percent of all injuries of the shoulder are recurrent injuries. If a player has sustained an injury of one shoulder, there is a high likelihood of that player sustaining an injury of the other shoulder also. Tackling is responsible for 49% of shoulder injuries during rugby.[1]

Posterior labral injuries may be increasingly common in rugby due to higher-impact tackles and increased training and competition demands. This may be similar to the findings described by Mair *et al*.[2] in their review of American football players in the 1990s, when the intensity with which the game was played and the tackling rules changed; they too reported a greater number of posterior labral injuries. Recent changes in the rules in the rugby union, with more open play and less stoppage of rucks and mauls, may also contribute to this.

Posterior labral injuries appear to be a prominent cause of posterior instability in patients with a significant traumatic episode.[7] However, not all our patients had true signs of posterior instability. Only 27% of our patients had a true dislocation and feelings of instability. The mechanism of injury in this study was similar in all cases and involved a direct blow to the anterior and lateral aspects of the shoulder while the arm was adducted. It is likely that this posteriorly directed force caused a posterior labral injury with subclinical instability and pain.[8]

This mechanism is similar to the shearing force mechanism described by Mair *et al*. in their series of nine American football athletes.[2] As opposed to the classical posteriorly directed force on the forward flexed, adducted, and internally rotated arm, a shear stress to the posterior labrum leads to isolated posterior labral injury, without involvement of the posterior capsule. This group of patients usually present with ongoing pain and reduced level of performance in their sports. This mechanism is also responsible for the 'dead arm' feeling described by the players at presentation, which can often be confused with 'stingers.'[7] All the patients had significant posterior pain in their shoulder during and after match play and, especially, during bench pressing. Only three patients had signs of posterior instability, which is similar to the findings of Mair *et al*.[2]

Early repair is recommended as conservative treatment has shown less promising results in similar groups of patients.Arthroscopic surgery has led to a better understanding of the various pathologies involved in posterior instability of the shoulder.[7,9] Arthroscopic stabilization, using suture anchors, for posterior shoulder instability is reported to be effective in athletes as far as providing pain relief, achieving stability, and restoring function are concerned.[9] This is also supported by our findings.

We have shown that operative intervention in this group of patients who are involved in a high-demand contact sport provides significant symptomatic relief, and permits early return to the preinjury level of function.

## CONCLUSION

Isolated traumatic posterior labral injury in the rugby player's shoulder is still not a widely recognized entity. There is a very specific mechanism of injury. These injuries can be extremely disabling for the elite rugby player. It is vital to have an early diagnosis and provide appropriate surgical treatment. Arthroscopic suture anchor repair and a supervised accelerated rehabilitation program gives successful clinical results, with rapid return to the sport.
